# Rapid Initiation of Intravenous Epoprostenol Infusion Is the Favored Option in Patients with Advanced Pulmonary Arterial Hypertension

**DOI:** 10.1371/journal.pone.0121894

**Published:** 2015-04-06

**Authors:** Mai Kimura, Yuichi Tamura, Makoto Takei, Tsunehisa Yamamoto, Tomohiko Ono, Masataka Kuwana, Keiichi Fukuda, Toru Satoh

**Affiliations:** 1 Department of Cardiology, Keio University School of Medicine, Tokyo, Japan; 2 Department of Rheumatology, Nippon Medical School, Tokyo, Japan; 3 Department of Cardiology, Kyorin University School of Medicine, Tokyo, Japan; Georgia Regents University, UNITED STATES

## Abstract

**Background:**

Intravenous infusion (IVI) of epoprostenol is an effective treatment for patients with advanced pulmonary arterial hypertension (PAH). However, there is no widely accepted standard method for initiating the IVI therapy. This study evaluated the hemodynamic improvements achieved with IVI epoprostenol to determine the optimal protocol for treatment initiation.

**Methods and Results:**

We retrospectively analyzed 42 consecutive PAH patients who underwent IVI epoprostenol in Keio University Hospital from 2001 to 2013. The study group comprised 30 women with a mean age of 34.3 ± 1.9 years. The etiology of PAH was idiopathic or heritable PAH (I/HPAH) in 38 cases, PAH associated with connective tissue disease in 3, and Eissenmenger’s syndrome in the remaining case. We divided the patients into rapid- and slow-initiation therapy groups according to the cumulative epoprostenol dose administered during the first 180 days, and compared the hemodynamic changes between the groups. The median cumulative doses were 6142 ± 165 μg/kg and 3998 ± 132 μg/kg epoprostenol, respectively. While there were no significant differences in mean pulmonary artery pressure (mPAP), pulmonary vascular resistance (PVR), or cardiac index (CI) between the groups before the IVI epoprostenol therapy, the rapid-initiation therapy group achieved significant improvements in these hemodynamic data compared with the slow-initiation therapy group (*P* < 0.005) at the follow-up right-heart catheterization (RHC).

**Conclusion:**

Rapid initiation of IVI epoprostenol therapy achieved the optimal hemodynamic improvements in patients with severe PAH.

## Background

Pulmonary arterial hypertension (PAH) is a rare, progressive, and fatal disease characterized by raised pulmonary vascular resistance (PVR), and resulting in right ventricular dysfunction due to increased right-heart afterload. Raised PVR is caused by pulmonary vasoconstriction, vascular remodeling of the small pulmonary arteries, and thrombosis [1]. In the absence of treatment, the median reported survival after diagnosis for idiopathic PAH (IPAH) is only 2.8 years [2].

Epoprostenol is a prostaglandin analogue that was first approved for patients with advanced PAH as a continuous intravenous infusion (IVI) in 1995 [3]. A 12-week, open, randomized, prospective study showed a positive effect of epoprostenol on survival in patients with New York Heart Association (NYHA) functional class III or IV IPAH [4]. Since then, many trials of epoprostenol have shown clinical and hemodynamic improvement with increased survival [5,6].

The dose of IVI epoprostenol is adjusted upward depending on the severity of PAH and side effects of the drug such as thrombocytopenia, hypotension, or high cardiac output. However, despite the many promising trial results with this drug, the optimal dose of IVI epoprostenol remains controversial. Some reports described the appropriate dose as 25 to 40 ng/kg/min [7–11], while others such as Akagi et al. [12] showed the efficacy of high-dose IVI epoprostenol. Moreover, there is no widely accepted standard method for initiating the IVI epoprostenol treatment, or any conclusive findings as to whether the increase in epoprostenol delivery should be rapid or slow with respect to eliciting improvements in PAH status. To address these important knowledge gaps, we evaluated the hemodynamic changes in PAH patients treated with IVI epoprostenol at our hospital, according to the original dose-up protocol, and we investigated the optimal protocol for initiating IVI epoprostenol.

## Methods

### Patients

This is a single-center, retrospective study. All PAH patients who received IVI epoprostenol at Keio University Hospital (Tokyo, Japan) from January 2001 to April 2013 were identified using a clinical database. This study was approved by the local ethical committee (KEIO UNIVERSITY SCHOOL OF MEDICINE AN ETHICAL COMMITTEE, Tokyo, Japan, approval number: 2010008). And written informed consent for their clinical records to be used in this study was given by all of the participants. In each patient, the IVI epoprostenol treatment was initiated according to the different protocols considered to be optimal at the time. Continuous infusion of IVI epoprostenol was delivered via a tunneled central venous catheter (Hick-mann’s catheter), inserted under fluoroscopic guidance. We enrolled all patients for whom we could obtain protocols for the initiation of IVI epoprostenol and who received follow up right-heart catheterization (RHC) in Keio University Hospital within several months after the initiation of IVI epoprostenol. Patient files and the clinical database were reviewed and data were collected on NYHA functional classification (FC) at the initial visit to our hospital, protocol of the IVI epoprostenol initiation, medication for pulmonary hypertension (phosphodiesterase type 5 inhibitors (PDE5i), endothelin receptor antagonist (ERA), prostanoids), hemodynamic data (mean pulmonary artery pressure (mPAP), pulmonary vascular resistance (PVR), and cardiac index (CI)) assessed by RHC, before and after the initiation of IVI epoprostenol. The NYHA FC of patients was allocated by the treatment physician, according to the WHO Functional Classification of PAH (whereby an FC of 1–4 is derived from patient symptoms in relation to exercise capacity).

### Group classification

The dose-up protocols of IVI epoprostenol were extensively reviewed, and the cumulative IVI epoprostenol dose for 180 days after initiation was determined. The patients were then divided into two groups according to the cumulative epoprostenol dose, with patients who had received 4700 μg/body weight [kg] or more during the initial 180 days classified into a rapid-initiation therapy group, and those who had received less than 4700 μg/body weight [kg] classified into a slow-initiation therapy group. Hemodynamic data assessed by RHC before and after the initiation of IVI epoprostenol were compared between the groups.

### Statistical analysis

We analyzed differences in the patient hemodynamic data (mPAP, PVR, CI) between the groups (rapid initiation therapy group and slow initiation therapy group). Data are presented as mean ± SEM. Mean PAP before and after the initiation of IVI epoprostenol exhibited normal distributions, so Student’s t-test was performed to analyze this data. Mann-Whitney’s U test was performed to analyze PVR and CI, both before and after the initiation of the therapy, since PVR and CI after the initiation of IVI epoprostenol did not exhibit normal distributions.

Two-sided *P*-values of less than 0.05 were considered to be statistically significant. Statistical analyses were performed with use of SPSS version 21.

## Results

### Patient enrollment

From January 2001 to June 2013, a total of 58 PAH patients received IVI epoprostenol treatment in Keio University Hospital. Four patients died soon after the initiation of IVI epoprostenol, three were loss to follow-up, three underwent lung transplantation in the United States, and another six were excluded due to missing protocols for the initiation of IVI epoprostenol.

**Fig 1:**
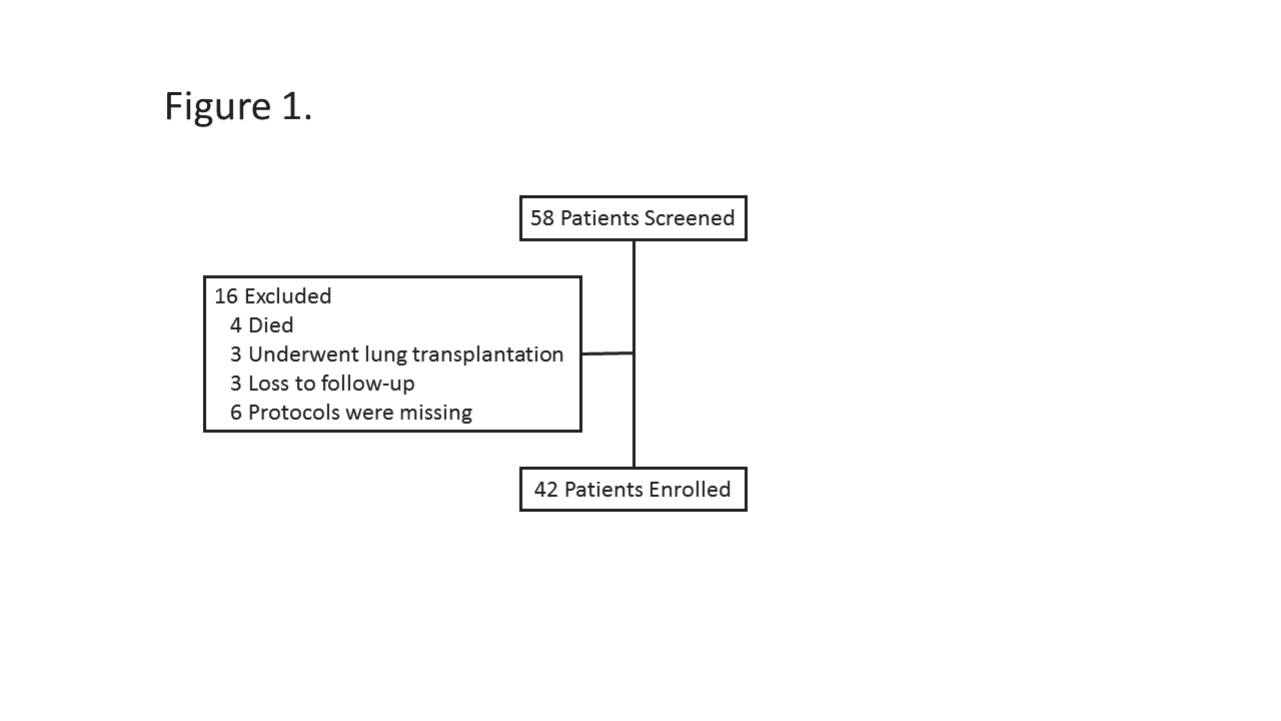
Patient inclusion. Flow chart describing patient inclusion protocol. Within the study period (2001–1013) 58 patients with PAH received IVI epoprostenol in Keio University hospital. Among the group, 16 patients were excluded from this study because they were lost to follow up, had missing protocols, died, or underwent a lung transplantation soon after the initiation of IVI epoprostenol.

Finally, we enrolled 42 patients, whose protocol for the initiation of IVI epoprostenol could be obtained and who received follow-up RHC within several months (Fig 1). The enrolled cohort comprised 30 women, with a mean age of 34.3 ± 1.9 years. The etiology of PAH was idiopathic PAH (IPAH) for 33 patients, heritable PAH (HPAH) for 5 patients, associated with connective tissue disease (CTD-PAH) for 3 patents, and Eissenmenger’s syndrome (ES) for 1 patient. Seven patients were classified as NYHA FC 2 at the first visit to our hospital, but their symptoms subsequently deteriorated, such that they became indicated for IVI epoprostenol treatment.

### Dosing of IVI epoprostenol

**Fig 2:**
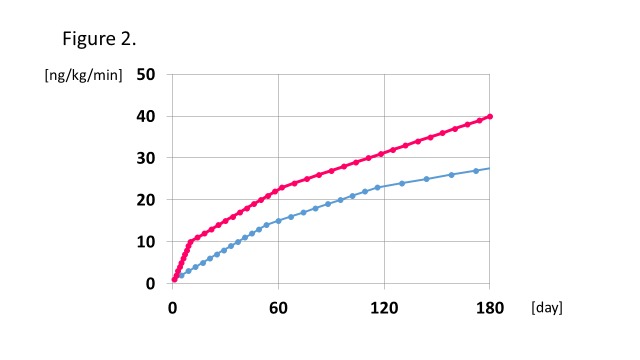
Typical protocols for rapid and slow initiation of therapy. The blue and red lines indicate the standard dosing schedules for the slow- and rapid-initiation IVI epoprostenol therapy, respectively.

**Fig 3:**
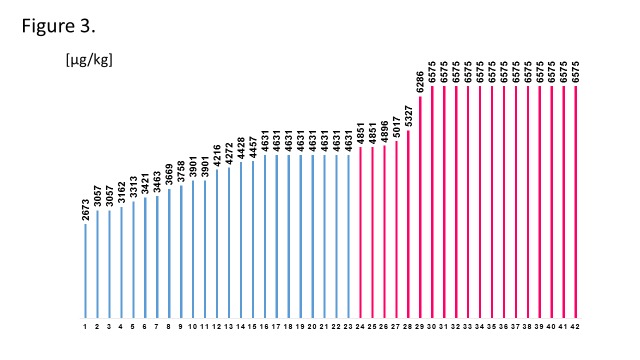
Cumulative epoprostenol dose for each patient. Bars show the cumulative dose of epoprostenol per body weight within the initial 180 days. The blue and red bars describe each patient’s cumulative dose in the slow- and rapid-initiation groups, respectively.

The initial dose of epoprostenol was 1 ng/kg/min for all patients, and then increased by 1 ng/kg/min every day, every several days, or weeks, according to each protocol. The typical protocols of both groups are shown in Fig 2. And the precise protocol of each patient is also shown in S1 Fig To compare the protocols, the cumulative IVI epoprostenol doses for 180 days after the initiation were calculated (Fig 3). The mean cumulative epoprostenol dose (± SE) during the initial 180 days was 4968 ± 136 mg/body weight [kg] (range 2673–6575 mg/body weight [kg]), and we classified them into two groups by the median value with 23 classified into the slow-initiation therapy group and 19 into the rapid-initiation therapy group. We used inotropic agents as appropriate at the beginning for severe patients with right heart failure, and no patients failed the intended protocol. The years when the patients initiated epoprostenol in each group are shown in S1 Table. Baseline characteristics of the two groups are shown in Table 1. More patients in the rapid-initiation therapy group were taking PDE5i and ERA than in the slow-initiation therapy group (p = 0.007, p = 0.042, respectively).

**Table 1:** Baseline clinical characteristics.

	Slow initiation therapy group (n = 23)	Rapid initiation therapy group (n = 19)	Total (n = 42)	*P* value*
**Age, yr**	35.2 ± 2.1	33.2 ± 3.5	34.3 ± 1.9	0.612
**Female sex, n (%)**	18 (78.2)	12 (63.1)	30 (71)	0.300
**Asian race, n (%)**	23(100)	19 (100)	42 (100)	-
**Body mass index, kg/m** ^**2**^	21.0 ± 0.8	20.2 ± 0.6	20.6 ± 0.5	0.356
**Etiology of PAH, n (%)**				
**IPAH**	17 (73.9)	16 (84.2)	33 (78.6)	0.431
**HPAH**	5 (21.7)	0 (0)	5 (11.9)	0.022
**CTD-PAH**	1 (4.3)	2 (10.5)	3 (7.1)	0.451
**ES**	0 (0)	1 (5.3)	1 (2.3)	0.331
**NYHA functional class at the first visit, n (%)**				
**Ⅱ**	3 (13.0)	3 (15.8)	6 (14.3)	0.814
**Ⅲ**	19 (82.6)	14 (73.7)	33 (78.6)	
**Ⅳ**	1 (4.3)	2 (10.5)	3 (7.1)	
**Medication**				
**PDE5i**	5 (21.7)	12 (63.1)	17 (40.4)	0.007
**ERA**	3 (13.0)	8 (42.1)	11 (26.1)	0.042
**Prostanoid**	6 (26.1)	5 (26.3)	11 (26.1)	0.987
**Warfarin**	7 (30.4)	5 (26.3)	12 (28.6)	0.775
**Duration of IVI epoprostenol, days**	240 ± 20	194 ± 12	219 ± 12	0.052
**Cumulative epoprostenol dose, μg/body weight [kg]**	3998 ± 132	6142 ± 165	4968 ± 136	< 0.001

Plus–minus values are means ± SE.

* *P* values indicate the statistical difference between slow and rapid initiation of therapy.

PAH, pulmonary arterial hypertension; IPAH, idiopathic pulmonary arterial hypertension; HPAH, heritable pulmonary arterial hypertension; CTD-PAH, PAH associated with connective tissue disease, ES, Eissenmenger’s syndrome; NYHA, New York Heart Association; PDE5i, phosphodiesterase type 5 inhibitor; ERA, endothelin receptor antagonist.

### Hemodynamic measurements

Follow up RHC was performed 219 ± 12 days (240 ±20 days for slow-initiation therapy group, and 194 ± 12 days for rapid-initiation therapy group) after the initiation of IVI epoprostenol. In the slow- and rapid-initiation groups at baseline, mPAPs were 64.7 ± 2.7 mmHg and 59.9 ± 3.6 mmHg, PVR were 23.7 ± 1.9 Wood’s Units and 18.5 ± 2.3 Wood’s Units, and CI were 1.73 ± 0.07 L/min/m^2^ and 1.97 ± 0.14 L/min/m^2^, respectively, whereas at the follow-up RHC, the comparative values were 53.4 ± 1.9 mmHg and 44.6 ± 2.8 mmHg, 14.4 ± 1.6 Wood’s Units and 7.9 ± 0.9 Wood’s Units, and 2.43 ± 0.18 L/min/m^2^ and 3.34 ± 0.33 L/min/m^2^, respectively.

**Fig 4:**
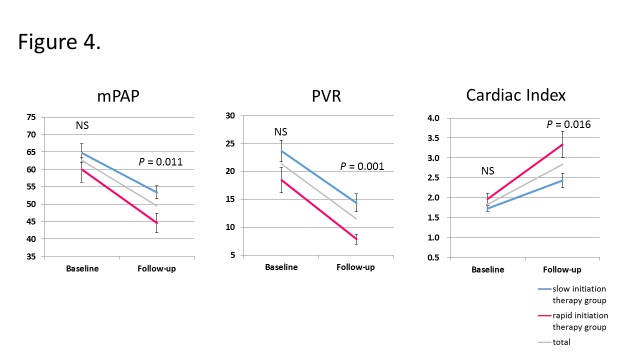
Improvements in hemodynamic data following IVI epoprostenol therapy. At follow up, the rapid-initiation group achieved significant improvements in mPAP, PVR and CI compared with the slow-initiation group, while there were no significant differences at baseline. mPAP: mean pulmonary artery pressure, PVR: pulmonary vascular resistance, CI: cardiac index, NS: not significant

As shown in Fig 4, mPAP, PVR, and CI were not significantly different between the groups before the initiation of IVI epoprostenol, whereas at the follow-up RHC, both mPAP and PVR were significantly decreased, and CI was significantly improved, in the rapid-initiation therapy group compared with the slow-initiation therapy group, suggesting a significant improvement in hemodynamic parameters with rapid initiation of IVI epoprostenol.

In order to make sure that the hemodynamic improvement was not achieved just by high dose of epoprostenol, but by rapid initiation, we compared the hemodynamic data of the groups one year after the initiation, when most of the patients reached to the final dose. One year follow up RHC was performed 381 ± 19 days (412 ± 124 days for slow-initiation therapy group, and 344 ± 29 days for rapid-initiation therapy group, p = 0.081). The mean values of mPAPs were 48.3 ± 2.4 mmHg and 40.9 ± 2.7 mmHg, PVR were 12.2 ± 1.3 Wood’s Units and 7.4 ± 0.8 Wood’s Units, and CI were 2.51 ± 0.16 L/min/m^2^ and 3.16 ± 0.23 L/min/m^2^ in the slow- and rapid-initiation groups, respectively. And as shown in S2 Fig, hemodynamic data in the rapid-initiation therapy group showed the significant improvement compared with the slow-initiation therapy group. On the other hand, the dosages of epoprostenol were not significantly different between the groups (35.1 ± 1.7 ng/kg/min for slow-initiation therapy group, and 39.5 ± 1.2 ng/kg/min for rapid-initiation therapy group, p = 0.063). Therefore, we concluded that the hemodynamic improvement of the rapid initiation therapy group was achieved by the initiation protocol, not by the dose of epoprostenol, and which brought long term improvements in hemodynamics.

### Subgroup analyses

A significantly larger number of patients in the rapid-initiation therapy group was taking PDE5i or ERA before the initiation of IVI epoprostenol compared to the slow-initiation therapy group, thus a subgroup comparison was conducted in the rapid-initiation therapy patients between those who taking none or one vasodilatory agent (PDE5i, ERA, or prostanoid) and those taking two or more agents at baseline.

As shown in Table 2, although both mPAP and PVR were significantly low, and CI was significantly high in those who took two or more agents at baseline, there were no significant differences in mPAP, PVR, or CI between the two groups at the follow-up RHC.

**Table 2:** Changes in hemodynamic data from baseline to follow-up right heart catheterization with rapid initiation therapy.

	Oral vasodilator agents		Total	*P* value*
	none or one (n = 11)	two or three (n = 8)		
**Baseline**				
**mPAP [mmHg]**	61.5 ± 5.0	57.8 ± 5.4	59.1 ± 3.5	0.616
**PVR [Wood’s Units]**	19.4 ± 3.3	17.3 ± 3.3	18.3 ± 2.2	0.717
**CI [L/min/m** ^**2**^ **]**	1.82 ± 0.19	2.17 ± 0.19	1.97 ± 0.13	0.238
**Follow-up**				
**mPAP [mmHg]**	44.7 ± 4.1	44.5 ± 3.8	44.2 ± 2.7	0.969
**PVR [Wood’s Units]**	8.1 ± 1.3	7.6 ± 1.2	7.7 ± 0.9	0.778
**CI [L/min/m** ^**2**^ **]**	3.48 ± 0.50	3.15 ± 0.37	3.41 ± 0.32	0.657

Plus–minus values are means ±SE.

* *P* values indicate the statistical difference between slow and rapid initiation of therapy.

mPAP, mean pulmonary arterial pressure; PVR, pulmonary vascular resistance; CI, cardiac index.

We also compared the hemodynamic data between the groups particularly in patients with IPAH and HPAH. As shown in S3 Fig, mPAP, PVR, and CI were not significantly different between the slow- and rapid-initiation groups before the initiation of IVI epoprostenol (mPAP were 64.8 ± 2.8 mmHg and 61.1 ± 4.0 mmHg, PVR were 23.5 ± 1.9 Wood’s Units and 19.3 ± 2.7 Wood’s Units, and CI were 1.73 ± 0.07 L/min/m^2^ and 1.97 ± 0.14 L/min/m^2^, respectively), whereas at the follow-up RHC, both mPAP and PVR were significantly decreased, and CI was significantly increased in the rapid-initiation therapy group compared with the slow-initiation therapy group (180 day follow up RHC; mPAP were 53.2 ± 2.0 mmHg and 44.2 ± 3.2 mmHg, PVR were 14.0 ± 1.6 Wood’s Units and 7.7 ± 1.0 Wood’s Units, and CI were 1.73 ± 0.07 L/min/m^2^ and 1.97 ± 0.14 L/min/m^2^, one year follow up RHC; mPAP were 48.2 ± 2.5 mmHg and 39.8 ± 2.9 mmHg, PVR were 12.2 ± 1.3 Wood’s Units and 7.4 ± 0.8 Wood’s Units, and CI were 2.51 ± 0.16 L/min/m^2^ and 3.16 ± 0.23 L/min/m^2^, respectively). These findings suggested that there was also the significant improvement in hemodynamic parameters with rapid initiation of IVI epoprostenol in the patients with IPAH/ HPAH.

## Discussion

This study showed the efficacy of rapid initiation of epoprostenol IVI therapy. Compared with the patients in the slow-initiation group (cumulative dose of epoprostenol less than 4700 μg/body weight [kg] in initial 180 days), patients receiving a rapid initiation of epoprostenol IVI enjoyed superior hemodynamic improvements in mPAP, CI, and PVR.

Although many oral agents for PAH have been approved, IVI epoprostenol is still the so-called "last line of defense for PAH treatment". In some clinical trials for IPAH or PAH associated with scleroderma, epoprostenol improves disease symptoms, exercise capacity, and hemodynamics [15,16]. Moreover epoprostenol is the only therapy that has achieved mortality reduction in a randomized study for IPAH [4].

A meta-analysis for total mortality of the three epoprostenol RCTs [4,13,14] showed a 70% relative risk reduction, and based on this the recent guidelines classify only epoprostenol as a Class I therapy in patients with severe (WHO-FC IV) PAH [15]. In addition, a recent report revealed a survival benefit of the upfront triple-combination therapy including IVI epoprostenol in patients with severe PAH [16].

The maximum dose-dependent epoprostenol efficacy in patients with PAH [12,17], and a dose-dependent reduction in PVR with epoprostenol IVI therapy [18] were previously reported in patients with PAH. However, there is no apparent evidence that the efficacy of epoprostenol also depends on the initiation schedule, despite anecdotal evidence from PAH specialists of the merits of rapid initiation. Accordingly, we aimed to design the best way to optimize the initiation of epoprostenol in order to achieve maximal hemodynamic improvement, and herein, confirmed the importance of rapid initiation of epoprostenol as well as high-dose usage in patients with severe PAH, which may also suggest further long-term survival benefit with such a protocol.

This study has some limitations. First, it was a single-center, retrospective study. As our institution is a center for PAH, some of the patients were diagnosed as IPAH in other hospitals and then referred to our center after initiations of various oral agents, possibly introducing a selection bias. Secondly, application of the initiation protocols differs from time to time. For instance, recent patients tend to undergo the rapid-initiation protocol and take oral combination therapies. Indeed, in this study, the number of patients who were taking PDE5i or ERA before the initiation of IVI epoprostenol was significantly larger in the rapid-initiation therapy group than in the slow-initiation therapy group. Therefore, we conducted subgroup analysis in the rapid-initiation group to compare between patients taking none or one vasodilatory agent and those taking two or more agents at baseline. However, there were no significant differences in any hemodynamic parameters (mPAP, PVR, or CI) between these groups at the follow-up RHC, suggesting that the improvements in hemodynamic data achieved by IVI epoprostenol exceeded the effect of concomitant medications. Finally, this study analyzed only a short period (180 days and one year) of data during the initiation of epoprostenol, and analysis of a possible association between long-term mortality and the cumulative dose of epoprostenol will be needed in the future.

In conclusion, compared with slow initiation, rapid initiation of IVI epoprostenol therapy significantly improved the hemodynamics in patients with PAH.

## Supporting Information

S1 Fig Precise protocols for all the enrolled patients. The blue and red lines indicate the dosing schedules for the patient classified into slow- and rapid-initiation therapy, respectively.
(TIF)

S2 Fig Improvements in hemodynamic data one year after the initiation of IVI epoprostenol therapy. At the time, rapid-initiation group showed significant improvements in mPAP, PVR, and CI compared with the slow-initiation group, as well as those of 180 days follow up RHC. mPAP: mean pulmonary artery pressure, PVR: pulmonary vascular resistance, CI: cardiac index, NS: not significant
(TIF)

S3 Fig Improvements in hemodynamic data 180 day and one year after the initiation of IVI epoprostenol therapy in patients of IPAH and HPAH (N = 38). The rapid-initiation group achieved significant improvements in mPAP, PVR and CI compared with the slow-initiation group in 180 days and one-year follow up RHC. mPAP: mean pulmonary artery pressure, PVR: pulmonary vascular resistance, CI: cardiac index, NS: not significant
(TIF)

S1 Table The number of patients whom enrolled slow- or rapid-initiation therapy protocols in each year. (DOC)
