# Supplementary figures and images for: Rapid Initiation of Intravenous Epoprostenol Infusion Is the Favored Option in Patients with Advanced Pulmonary Arterial Hypertension
Source: PLoS One. 2015 Apr 6;10(4):e0121894. doi: 10.1371/journal.pone.0121894 (PMC4386822; doi:10.1371/journal.pone.0121894)

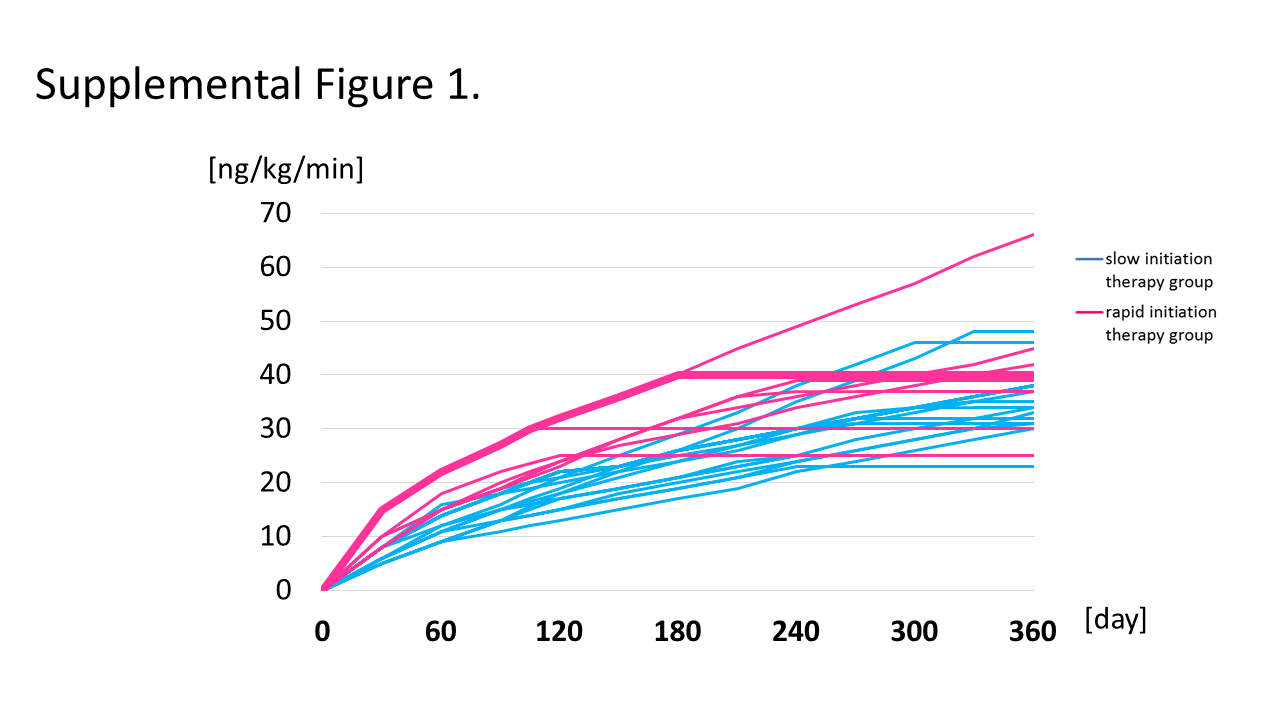

Supplement: S1 Fig — The blue and red lines indicate the dosing schedules for the patient classified into slow- and rapid-initiation therapy, respectively. (TIF) [file pone.0121894.s001.tif]

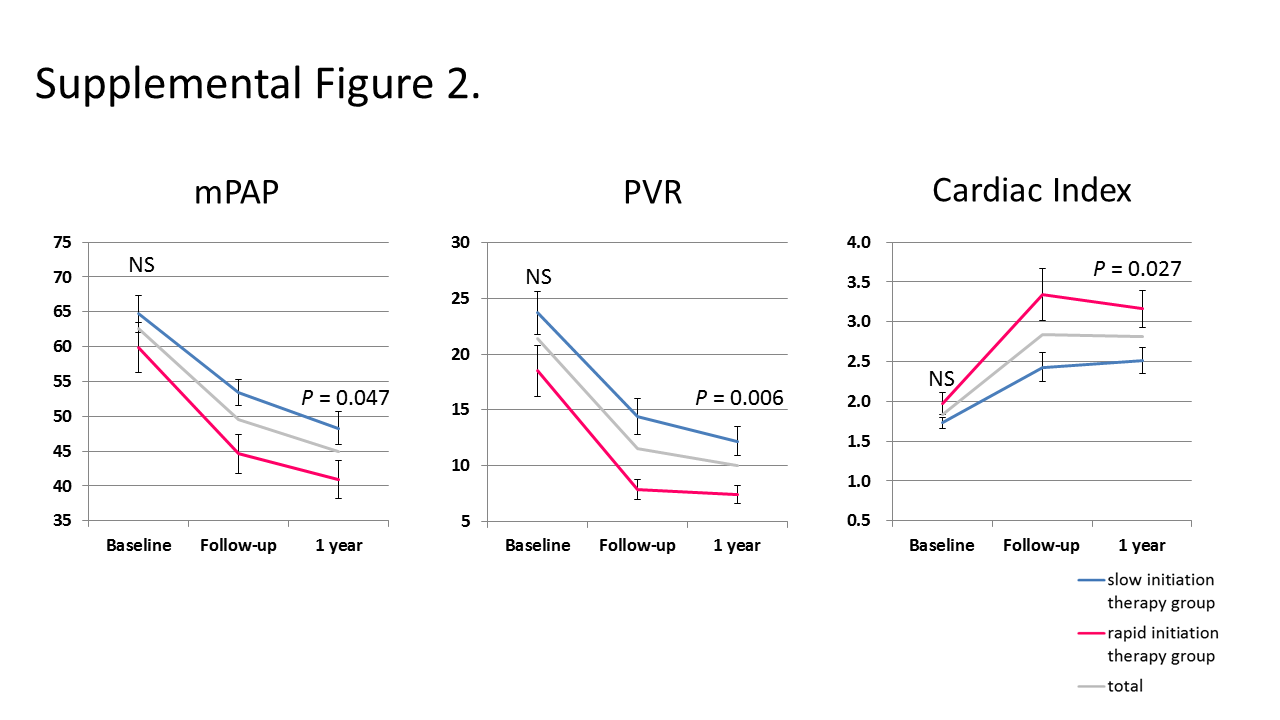

Supplement: S2 Fig — At the time, rapid-initiation group showed significant improvements in mPAP, PVR, and CI compared with the slow-initiation group, as well as those of 180 days follow up RHC. mPAP: mean pulmonary artery pressure, PVR: pulmonary vascular resistance, CI: cardiac index, NS: not significant (TIF) [file pone.0121894.s002.tif]

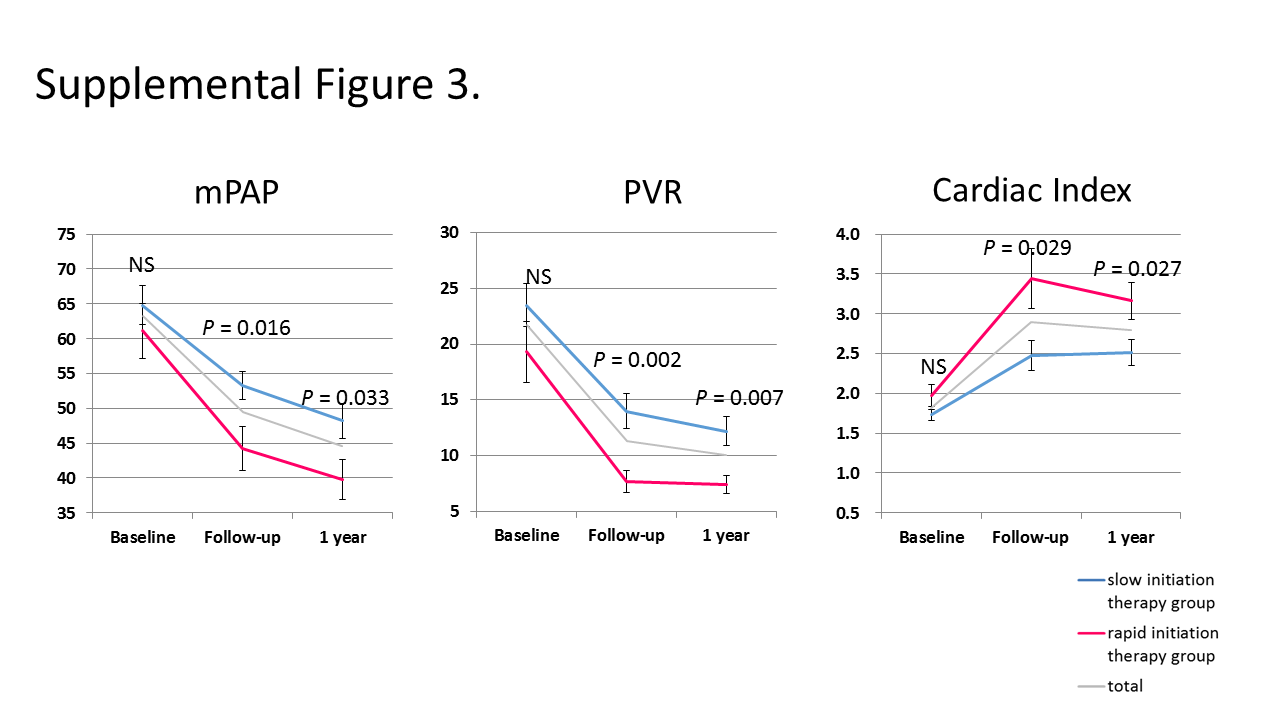

Supplement: S3 Fig — The rapid-initiation group achieved significant improvements in mPAP, PVR and CI compared with the slow-initiation group in 180 days and one-year follow up RHC. mPAP: mean pulmonary artery pressure, PVR: pulmonary vascular resistance, CI: cardiac index, NS: not significant (TIF) [file pone.0121894.s003.tif]
