# Supplementary material for: Rapid Initiation of Intravenous Epoprostenol Infusion Is the Favored Option in Patients with Advanced Pulmonary Arterial Hypertension
Source: PLoS One. 2015 Apr 6;10(4):e0121894. doi: 10.1371/journal.pone.0121894 (PMC4386822; doi:10.1371/journal.pone.0121894)
Supplement: S1 Table — (DOC) [file pone.0121894.s004.doc]

**Rapid initiation of intravenous epoprostenol infusion is the favored option in patients with advanced pulmonary arterial hypertension**

Mai Kimura, MD, Yuichi Tamura, MD, PhD, Makoto Takei, MD, Tsunehisa Yamamoto, MD, Tomohiko Ono, MD, Masataka Kuwana, MD, PhD, Toru Satoh, MD, PhD, Keiichi Fukuda, MD, PhD

**Supplemental Table 1.**

**The numbers of the patients initiated epoprostenol therapy in each groups**

|  | **Slow-initiation therapy group (n)** | **Rapid-initiation therapy group (n)** |
| --- | --- | --- |
| **2001** | **3** | **2** |
| **2002** | **3** | **0** |
| **2003** | **1** | **0** |
| **2004** | **4** | **0** |
| **2005** | **4** | **1** |
| **2006** | **0** | **0** |
| **2007** | **6** | **1** |
| **2008** | **0** | **2** |
| **2009** | **1** | **0** |
| **2010** | **0** | **5** |
| **2011** | **0** | **2** |
| **2012** | **0** | **5** |
| **2013** | **0** | **2** |
